# Review of Mendelian Randomization Studies on Endometrial Cancer

**DOI:** 10.3389/fendo.2022.783150

**Published:** 2022-05-09

**Authors:** Jian-Zeng Guo, Qi-Jun Wu, Fang-Hua Liu, Chang Gao, Ting-Ting Gong, Gang Li

**Affiliations:** ^1^ Department of Clinical Epidemiology, Shengjing Hospital of China Medical University, Shenyang, China; ^2^ Department of Obstetrics and Gynecology, Shengjing Hospital of China Medical University, Shenyang, China; ^3^ Clinical Research Center, Shengjing Hospital of China Medical University, Shenyang, China; ^4^ Department of Ultrasound, Shengjing Hospital of China Medical University, Shenyang, China

**Keywords:** causality, instrumental variables, Mendelian randomization, endometrial cancer, risk factors

## Abstract

Endometrial cancer (EC) is a common gynecological cancer. In some parts of the world, the incidence and mortality of EC are on the rise. Understanding the risk factors of EC is necessary to prevent the occurrence of this disease. Observational studies have revealed the association between certain modifiable environmental risk factors and EC risk. However, due to unmeasured confounding, measurement errors, and reverse causality, observational studies sometimes have limited ability to judge robust causal inferences. In recent years, Mendelian randomization (MR) analysis has received extensive attention, providing valuable insights for cancer-related research, and is expected to identify potential therapeutic interventions. In MR analysis, genetic variation (alleles are randomly assigned during meiosis and are usually independent of environmental or lifestyle factors) is used instead of modifiable exposure to study the relationship between risk factors and disease. Therefore, MR analysis can make causal inference about exposure and disease risk. This review briefly describes the key principles and assumptions of MR analysis; summarizes published MR studies on EC; focuses on the correlation between different risk factors and EC risks; and discusses the application of MR methods in EC research. The results of MR studies on EC showed that type 2 diabetes, uterine fibroids, higher body mass index, higher plasminogen activator inhibitor-1 (PAI-1), higher fasting insulin, early insulin secretion, longer telomere length, higher testosterone and higher plasma cortisol levels are associated with increased risk of EC. In contrast, later age of menarche, higher circulatory tumor necrosis factor, higher low-density lipoprotein cholesterol, and higher sex hormone-binding globulin levels are associated with reduced risk of EC. In general, despite some limitations, MR analysis still provides an effective way to explore the causal relationship between different risk factors and EC.

## Introduction

According to the 2020 global cancer statistics, endometrial cancer (EC) accounts for 4.5% of female tumors (1, 2). An estimated 417,367 new cases and 97,370 deaths were attributed to EC globally (1–3). Although the surgical treatment of EC has been refined, challenges still exist. Reports in recent years have pointed out that, unlike most other cancers in the United States and several European countries, the incidence and mortality of EC are on the rise (3, 4). Therefore, it is necessary to understand the risk factors of EC. Epidemiological studies have shown that obesity and metabolic-related diseases, including diabetes and polycystic ovary syndrome, are risk factors for EC (5–7). In addition, too much estrogen also makes women susceptible to EC (8, 9). Conversely, factors that provide protection from EC include higher parity and the use of oral contraceptives (10, 11). Changing lifestyle and diet through public health measures is expected to have a significant impact on the incidence of EC.

The premise of the public health measure is to clarify the causal relationship between exposure and disease risk. In past studies, randomized controlled trials (RCT) are the gold standard for inferring the causal relationship between exposure and disease. However, they are often very expensive, time-consuming, and have a high failure rate (>50% due to lack of efficacy) (12, 13). Moreover, certain phenotypes (such as disease history) cannot be randomized in RCT. In addition, the sample size in RCT is relatively small (14–16). Therefore, observational study becomes another option to clarify the relationship between exposure and disease. Observational study refers to a type of research in which the researcher does not take action on the research participants, but observes the natural relationship between factors and outcomes (17). This type of research provides a wealth of information about the link between disease exposure and outcome. However, observational research is often difficult to avoid the influence of confounding factors and reverse causality (18). Confounding factors refer to all factors (including known and unknown) that may affect the outcome in addition to research factors (14, 15, 19). Reverse causality refers to the reversal of the order in observational studies due to the inability to accurately determine the chronological order of exposures and outcomes (20). Compared with RCT, observational studies rarely justify causal conclusions, even when there is a strong statistical association between exposure and outcome, because it is not certain that all confounders of the association have been identified, measured, and appropriately adjusted (21, 22).

In order to overcome the limitations of RCT and observational studies, Mendelian randomization (MR) is widely used as a method to study the causal relationship between exposure and disease. In the past few years, along with the experimental design of genome-wide association studies (GWAS), researchers have made many scientific and biological discoveries. These studies are designed to detect genomic locus variation associated with complex traits in the population, especially the detection of the association between common single nucleotide polymorphisms (SNPs) and common diseases (23). In order to examine the causal effects of these exposures on health outcomes (disease incidence or progression), MR uses germline genetic variation as an instrumental variable (IV), usually SNPs, to simulate the effects of modifiable exposures (e.g., environmental factors, biological traits, or drug pathways) on disease susceptibility (24, 25). In MR studies, researchers initially identify and extract information for SNPs associated with exposure at the genome-wide significance level (P=5×10^ (–8) and subsequently evaluate the relationship between these SNPs and outcomes to obtain odds ratios (OR) and mean differences. When the association between the exposure and the outcome is statistically significant, the exposure is determined to have a causal relationship with the outcome (26). Compared with observational research, the advantages of MR are mainly reflected in the following aspects. First, alleles are randomly allotted during meiosis, and are often independent from environmental or lifestyle factors. Second, with the continuous development of sequencing technology and analysis technology, in most cases, genetic variation can be accurately measured and reported. These genetic variants are sometimes associated with the representation of lifetime exposure which is particularly useful for assessing long-term risk factors (such as smoking, drinking, and chronic diseases) (27). In summary, MR provides another way to explore causality in epidemiological research. Correlations between exposure and outcome were measured using appropriate instrumental variables, and methodological rigor was improved by testing and adjusting for heterogeneity (22).

**Figure 1:**
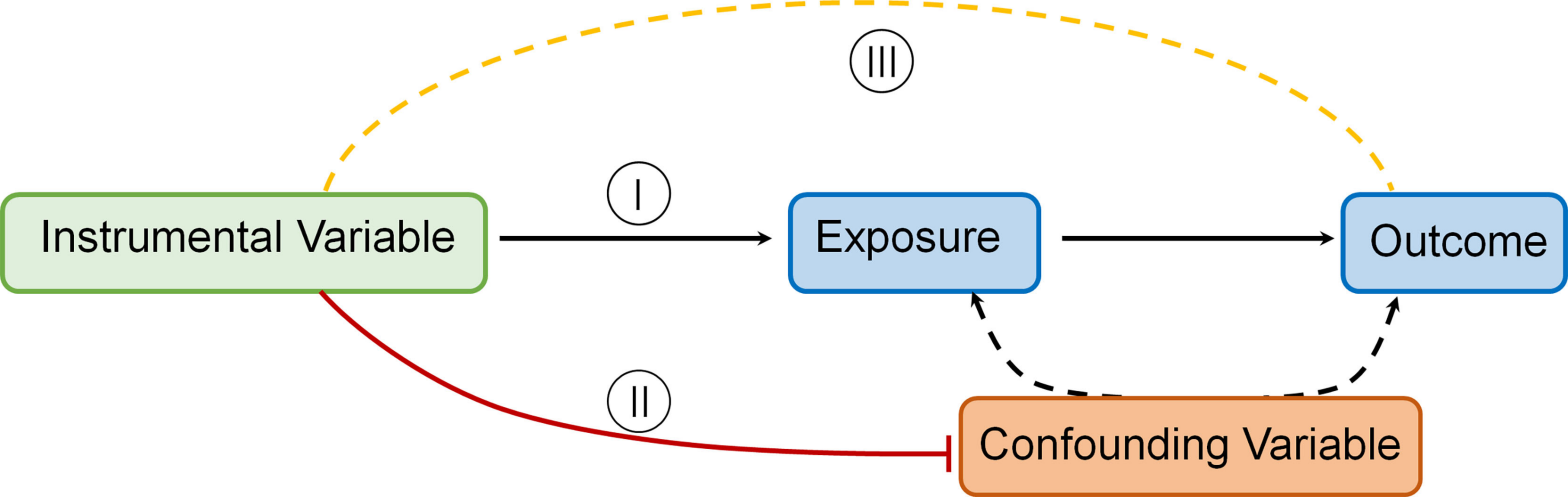
Directed acyclic graph depicting MR principles and underlying IV assumptions (I–III).

The MR technique relies on a number of assumptions for accuracy. The rationale underlying MR and required IV assumptions are as follows [**Figure 1** Directed acyclic graph depicting MR principles and underlying IV assumptions (I–III)]:

- IVs (SNPs being used) should be strongly linked to the exposure(s) in question.
- IVs should not be linked in any way to confounding variables.
- IVs should be linked to outcomes only through the exposure(s) in question.
- To estimate a causal effect with IV analysis, additional assumptions are required. One such assumption is that:
- The associations are linear and not affected by statistical interactions (14).

On this basis, there are also many statistical methods applied in the MR analysis process. The ratio of coefficients or Wald method is a direct and commonly used method of performing MR (28). The causal effect of exposure on outcome is derived from the ratio of the regression coefficient of the instrumental variable on the outcome to that of instrumental variable on the exposure (28). Two-stage least squares is another way to perform MR analysis. This method involves two stages of regression: the first is from instrumental variables to exposure, and the second is from exposure to outcome. This method requires individual-level data and biases when using at least one invalid instrumental variable (29). In addition, In order to make the standard error in the instrumental variable-result regression smaller, the inverse variance weighting method that gives SNPs higher weight is widely used in today’s MR research (30).

Nonetheless, there are some limitations that need to be considered in MR analysis. One of the common problems is horizontal pleiotropy. Horizontal pleiotropy indicates that the instrumental variable is not directly related to the result of exposure, which violates the third hypothesis of instrumental variables, and it is difficult to avoid in MR research (31). For the horizontal pleiotropy of one-sample MR, the Q test has a good test effect, especially when the data set is large (32). Another method that serves as a sensitivity analysis is an adaptation of Egger regression called MR-Egger, which can be used to detect bias due to horizontal pleiotropy (33). Linkage disequilibrium (defined as a non-random association between alleles at genetic locus on a chromosome) is also a common phenomenon. When the SNP used as IV is in linkage disequilibrium with the SNP that independently affects the outcome through exposure, it may violate the basic MR assumption (14). The Bayesian test that can be used to determine whether the association is the result of a colocalized SNP may reduce the linkage disequilibrium bias in MR analysis (34). As well as setting a maximum pairwise linkage disequilibrium threshold for SNP inclusion, methods such as penalized logistic regression have been described as a means of selecting SNPs based on the knowledge of linkage disequilibrium (35). In addition to the two points mentioned above, the “winner’s curse” phenomenon sometimes occurs in single-sample MR. Winner’s curse usually refers to a situation in which only the main SNPs with the smallest P value are reported, but other important SNPs are ignored or may not be mentioned (36). Two-sample MR analysis can solve this problem well (37).

## Application of MR in EC

Although epidemiological studies have revealed a large number of exposures related to increased or decreased EC risk, the causal relationship between these exposures and changes in EC risk has remained largely unclear. In the past few decades, it has become easier to identify genetic variants associated with many potential risk factors for health-related outcomes, relying on GWAS. The increasing number of GWAS results has promoted the use of MR in assessing the causal relationship between modifiable exposures and outcomes. In recent years, some MR research results focused on EC have also been published. In addition, the development of new methods in MR research has challenged the previously reported causal relationship between certain biomarkers and disease risk. Therefore, it is very important to record the progress of MR research and pay attention to the quality and effectiveness of MR. In this review, we have formulated strict literature retrieval strategies and selection criteria, sorted out and analyzed the MR studies on EC that have been published in the past, focusing on its advantages and limitations.

### Search Strategy and Selection Criteria

Original studies were identified by searching for relevant articles up to July 25, 2021, in the PubMed database. The search algorithms for PubMed database were as follows: “Mendelian randomization” or “genetic instrumental variable” or a related term (e.g., “genetic instrument”) and “Endometrial Cancer” or “Endometrial Neoplasm” or “Neoplasm, Endometrial” or “Endometrial Carcinoma” or “Cancer of Endometrium” or “Carcinoma of Endometrium”, with no restriction on subheadings. All retrieved articles were checked for relevant citations and studies not included in the above electronic sources were searched manually. We included studies which uses MR methodology and instrumental variable analysis to evaluate risk factors of EC. The search strategy and selection criteria have been checked by two independent authors and if necessary, the inconsistent part would be judged by third author. A total of 21 articles were finally included and classified according to type of exposure (**Table 1**).

**Table 1:** Mendelian randomization studies on endometrial cancer.

Author [ref], year	Exposure	Outcome	Sample size for the outcome data		Sources	SNPs	Estimate (95% CI)	MR methods
			Cases	Control				
Prescott et al, 2015 (38)	BMI	EC	3376	3867	Epidemiology of Endometrial Cancer Consortium	97	1.13 (1.04 to 1.22)	Pooled unconditional logistic regression
Nead et al, 2015 (39)	T2D	EC	1287	8273	Studies from the UK and Australia	49	0.91 (0.79 to 1.04)	IVW
	Fasting glucose					36	1.00 (0.67 to 1.50)	
	Fasting insulin					18	2.34 (1.06 to 5.14)	
	Early insulin secretion					17	1.40 (1.12 to 1.76)	
	BMI					32	3.86 (2.24 to 6.64)	
Painter et al, 2016 (40)	BMI	EC	6609	37926	ECAC	77	2.11 (1.94 to 2.28)	IVW
		Endometrioid EC					2.27 (2.08 to 2.45)	
	Waist-hip ratio	EC				47	0.97 (0.63 to 1.31)	
Thompson et al, 2016 (41)	Estradiol	EC	6608	37925	ECAC	105	1.09 (1.03 to 1.21)	
Day et al, 2017 (42)	Age at menarche	EC	4401	28758	ECAC	375	0.781 (0.699 to 0.872)	IVW
Haycock et al, 2017 (43)	Telomere Length	EC	6608	37925	ECAC	12	1.31 (1.07 to 1.61).	IVW
Kho et al, 2019 (44)	Selenium	EC	12906	108979	ECAC	4	0.99 (0.87 to 1.14)	Wald-type ratios/IVW
Ong et al, 2019 (45)	Coffee	EC	373	85999	UK Biobank	4	0.963 (0.912 to 1.018)	IVW
Yuan et al, 2020 (46)	TNF	EC	1520	366123	UK Biobank	3	0.25 (0.07 to 0.94)	IVW
Dimou et al, 2020 (47)	Adiponectin	EC	12906	108979	ECAC	18	1.02 (0.89 to 1.17)	IVW
	Leptin					2	1.46 (0.69 to 3.06)	
	sOB-R					4	1.02 (1.00 to 1.05)	
	PAI-1					4	1.38 (1.04 to 1.82)	
Ruth et al, 2020 (48)	Testosterone	EC	12,270	46,126	ECAC	254	1.39 (1.26 to 1.53)	IVW
		Endometrioid EC					1.39 (1.24 to 1.55)	
		Non-endometrioid EC					1.26 (0.99 to 1.61)	
	Sex hormone-binding globulin	EC				359	0.77 (0.67 to 0.89)	
		Endometrioid EC					0.78 (0.67 to 0.91)	
		Non-endometrioid EC					0.78 (0.55 to 1.11)	
Yuan et al, 2020 (49)	T2D	EC	1931	292606	UK Biobank	399	1.08 (1.01 to 1.15)	IVW
Masuda et al, 2021 (50)	BMI	EC	909	39556	BioBank Japan Project	74	1.22 (1.08 to 1.38)	IVW
			194174		UK Biobank	131	1.0008 (1.0002 to 1.0014)	
Kho et al, 2021 (51)	LDL cholesterol	EC	12906	108979	ECAC	141	0.90 (0.85 to 0.96)	IVW
		Endometrioid EC	8758	46126		142	0.93 (0.87 to 1.01)	
		Non-endometrioid EC	1230	35447		144	0.76 (0.63 to 0.90)	
	HDL cholesterol	EC	12906	108979		168	1.06 (0.99 to 1.13)	
		Endometrioid EC	8758	46126		168	1.02 (0.95 to 1.10)	
		Non-endometrioid EC	1230	35447		169	1.20 (1.00 to 1.45)	
	Triglycerides	EC	12906	108979		114	0.98 (0.90 to 1.06)	
		Endometrioid EC	8758	46126		115	0.96 (0.87 to 1.05)	
		Non-endometrioid EC	1230	35447		116	1.15 (0.90 to 1.45)	
Ahmed et al, 2021 (52)	Adiposity	EC	1208	145748	UK Biobank	127	1.77 (1.16 to 2.68)	IVW
Freuer et al, 2021 (53)	BMI	EC	12270	46126	ECAC, E2C2	297	1.75 (1.57 to 1.95)	IVW
	AFR					116	1.43 (1.24 to 1.65)	
	TFR					202	1.01 (0.92 to 1.11)	
	LFR					166	0.99 (0.93 to1.03)	
Larsson et al, 2021 (54)	Plasma cortisol	EC	12906	108979	ECAC	3	1.50 (1.13 to 1.99)	IVW
			879		FinnGen consortium			
Mullee et al, 2021 (55)	Total testosterone	EC	12906	108979	ECAC		1.38 (1.22 to 1.57)	IVW
	Free testosterone						2.07 (1.66 to 2.58)	
	SHBG						0.76 (0.67 to 0.86)	
	IGF-1						0.98 (0.90 to 1.07)	
Larsson et al, 2021 (56)	Endogenous 17β-estradiol	EC	12906	108979	ECAC	5	1.09 (1.06 to 1.11)	IVW
		Endometrioid EC	8758	46126			1.10 (1.07 to 1.13)	
		Non-endometrioid EC	1230	35447			1.02 (0.96 to 1.08)	
Kho et al, 2021 (57)	Endometriosis	EC	12270	46426	ECAC	26	1.09 (0.92 to 1.31)	IVW
	PCOS					14	0.95 (0.88 to 1.03)	
	Uterine fibroids					23	1.19 (1.03 to 1.36)	
O’Mara et al, 2021 (58)	BMI	EC	12906	108979	ECAC	77	1.92 (1.63 to 2.25)	IVW
		Endometrioid EC	8758	46126			2.04 (1.69 to 246)	
		Non-endometrioid EC	1230	35447			1.65 (1.13 to 2.41)	
	Waist:hip ratio	EC	12906	108979		47	0.95 (0.72 to 1.25)	
		Endometrioid EC	8758	46126			0.94 (0.71 to 1.24)	
		Non-endometrioid EC	1230	35447			1.27 (0.69 to 2.33)	
	Age at menarche (years); total effect	EC	12906	108979		368	0.82 (0.77 to 0.87)	
		Endometrioid EC	8758	46126			0.80 (0.74 to 0.86)	
		Non-endometrioid EC	1230	35447			0.93 (0.79 to 1.08)	
	Age at menarche (years); direct effect	EC	12906	108979		368	0.88 (0.82 to 0.94)	
		Endometrioid EC	8758	46126			0.86 (0.79 to 0.93)	
		Non-endometrioid EC	1230	35447			0.97 (0.82 to 1.16)	
	Age at natural menopause (years)	EC	12906	108979		54	1.03 (1.00 to 1.06)	
		Endometrioid EC	8758	46126			1.02 (0.99 to 1.06)	
		Non-endometrioid EC	1230	35447			1.07 (0.99 to 1.14)	
	Height	EC	12906	108979		814	1.00 (0.95 to 1.06)	
		Endometrioid EC	8758	46126			0.99 (0.93 to 1.05)	
		Non-endometrioid EC	1230	35447			1.00 (0.88 to 1.15)	

SNPs, single nucleotide polymorphisms; BMI, body mass index; EC, endometrial cancer; T2D, type 2 diabetes mellitus; IVW, Inverse-variance weighted; ECAC, Endometrial cancer Association Consortium; PAI-1, plasminogen activator inhibitor-1; sOB-R, soluble leptin receptor; LDL, low-density lipoprotein; HDL, high-density lipoprotein; AFR, arm fat ratios; TFR, trunk fat ratios; LFR, leg fat ratios; IGF-1, insulin-like growth factor-1; PCOS, polycystic ovary syndrome.

### Causality Between Obesity and EC Risk

Obesity, as defined in adults by a body mass index (BMI) of greater than or equal to 30 kg/m^2^, is a growing public health problem worldwide (59, 60). Over the past few decades, the prevalence of adult overweight and obesity has increased by 27% worldwide (61). Studies have shown that the health risks brought by obesity are related to the increased risk of a variety of diseases, including hypertension, type 2 diabetes, cardiovascular disease, osteoarthritis, renal failure, liver disease, and many types of cancer (62). Recent data indicate that the main pathways linking obesity and cancer include: 1) hyperinsulinemia and abnormal insulin-like growth factor-I (IGF-I) systems and signals; 2) sex hormone biosynthesis and pathways; 3) chronic low-grade inflammation and oxidative stress; 4) pathophysiological changes of adipocytes; 5) microenvironment and cell disturbance; 6) disorders of circadian rhythm and dietary nutrients; and 8) changes in the intestinal microbiome (63). Obesity is also a known risk factor for EC. An observational study showed that obesity (defined as BMI> 30 and <35 kg/m^2^) is associated with a 2.6-fold increase in the risk of EC, while severe obesity (BMI> 35 kg/m^2^) is associated with a 4.7-fold increase in the risk of EC (60).

The earliest MR study on obesity and EC was published in 2015. The results showed that in women of European descent, having a large number of BMI risk alleles increase the risk of EC caused by overweight (38). Furthermore, a MR study published in 2016 showed that genetic liability to obesity measured as BMI, but not waist: hip ratio, is causal for EC (40). Further analysis of subtype specificity showed that similar associations were found in endometrioid and non-endometrioid EC (40). Similarly, the results of an MR study on diabetes and EC in British and Australian women published in 2015 by Nead et al. showed that an increase in BMI was positively correlated with an increased risk of EC (39). Another MR study published subsequently found that obesity is a risk factor for EC in Japanese women (50). Three MR studies on BMI and EC published in 2021 all showed the same results (52, 53, 58). It is worth mentioning that the study by Freuer et al. pointed out that indicate evidence for arm fat, but not trunk or leg fat, as an EC risk factor (53).

### Causality Between Obesity-Related Factors and EC Risk

In addition to BMI, there have been some research results on obesity-related factors and EC. This article mainly discusses the following, including: adiponectin, leptin, soluble leptin receptor(sOB-R), plasminogen activator inhibitor-1(PAI-1), tumor necrosis factor (TNF), insulin-like growth factor-1(IGF-1), cholesterol and triglycerides. Visceral fat is composed of adipocytes and preadipocytes, as well as infiltrating macrophages, stroma, nerves and stem cells. Together, they secrete a series of adipokines, which exert local and systemic effects, increase endometrial proliferation and promote tumorigenesis (64). Estrogens and proinflammatory adipokines stimulate cell proliferation as seen in endometrial hyperplasia and carcinoma (65). Obesity-related pro-inflammatory adipokines, such as leptin, interleukin 6 and tumor necrosis factor a, inhibit normal insulin signaling, leading to insulin resistance and promoting endometrial proliferation (66–68). In addition, experimental studies have also shown that dietary lipids, including saturated fatty acids, unsaturated fatty acids and cholesterol intake, may affect EC risk by regulating the production, metabolism and excretion of endogenous hormones (69, 70). The results of a number of meta-analysis showed that adiponectin was negatively correlated with EC risk, while leptin was the opposite. There was no significant correlation between TNF, IGF-1 and EC risk (71–73). The results of the meta-analysis also support the association between PAI-1 4G/5G polymorphism and increased cancer risk. Especially among white people, people with 4G alleles have a high risk of endometrial cancer (74). Meta-analyses based on retrospective studies of dietary cholesterol and EC risk points out that case-control data indicate that total fat, saturated fat, and animal fat are associated with increased risk (70, 75). However, the limited available cohort study data does not support these associations. Before reaching a conclusion, additional data is needed, especially data from prospective studies. Nevertheless, in order to better prevent and treat EC, more research is still needed to clarify the causal link between obesity and EC.

The relationship between obesity-related factors and EC risk has also received extensive attention from MR research. An MR study on circulating adipokines concentration and the risk of five obesity-related cancers published in 2020 pointed out that adiponectin has no impact on the risk of EC (47). Similarly, leptin and sOB-R are also not related to the risk of EC (47). It is worth noting that the concentration of PAI-1 is positively correlated with the risk of EC (47). This association is only driven by the rs11128603 variant, which is also related to type 2 diabetes, obesity, and body characteristics (47). Consistent with the results of the observational meta-analysis, the results of the MR analysis showed that there was no correlation between IGF-1 levels and EC risk (55, 71). For TNF, MR analysis and meta-analysis showed different results. An MR study on TNF and disease risk published in 2020 pointed out that genetically predicted higher TNF levels are associated with a lower risk of EC (46). Recently, Kho et al. conducted an MR study on cholesterol and EC risk in women of European descent (51). The results showed that genetically raised low-density lipoprotein cholesterol levels were associated with lower risks of EC, regardless endometrioid and non-endometrioid subtypes (51). Conversely, higher high-density lipoprotein cholesterol levels were associated with increased risk of non-endometrioid EC (51). After accounting for the potential confounding role of obesity (as measured by genetic variants associated with BMI), the association between genetically predicted increased low-density lipoprotein cholesterol levels and lower EC risk remained significant, especially for non-endometrioid EC (51).

### Causality Between Height and EC Risk

Body development requires proliferation pathways that control cell metabolism and tissue growth, as well as selective “invasive” cell migration for organogenesis. These requirements are quite similar to the process of tumor growth and malignant transformation (76, 77). Height is also considered a potential risk factor for the development of endometrial cancer. There are meta-analysis results showing a positive correlation between height and EC risk (78). Meanwhile, human height-associated loci have been recently identified by genome-wide association studies (79). Strikingly, most of the more than 100 height-related genes found appear to be related to tumor growth and increase the risk of cancer (77). Therefore, researchers have carried out many MR studies on height and cancer. However, we only found one MR study on EC, which did not find an association between height and EC risk (58).

### Causality Between Sex Hormones and EC Risk

As early as the 1990s, there were researches on sex hormones and cancer (80). Although endogenous estrogen often has beneficial effects(such as regulating menstrual cycle, reproduction, bone density, brain function and cholesterol mobilization), continuous exposure to high levels of estrogen is widely regarded as a risk factor for various cancers, especially EC (81–83). After estrogen binds to the receptor, it can directly regulate the transcription of a variety of proliferation genes, and then stimulate the proliferation of the endometrium by activating the MAPK and AKT signaling pathways (84). In addition, estrogen acts not only as a mitogen, but also as a mutagen. The genotoxic metabolites of estrogen can react with DNA to form apurinic adducts, which eventually lead to the accumulation of double-stranded DNA breaks and lead to genetic instability (85–87). Testosterone is an essential hormone for women, with physiological actions mediated directly or *via* aromatisation to estradiol throughout the body (88). Prospective analyses of testosterone and sex hormone binding globulin (SHBG) with the risk of 19 types of cancer in men and postmenopausal women in UK Biobank have shown that free and total testosterone were associated with higher risk of EC in postmenopausal women, while SHBG were associated with lower risk (89). Previous small-scale studies have shown that there is no statistically significant positive correlation between circulating total testosterone or free testosterone concentration and the risk of endometrial cancer after menopause (90, 91). In female-to-male transsexuals, testosterone is antiproliferative in the endometrium, with no evidence of endometrial proliferation in a RCT of testosterone done over 12 months (92). In addition, it has also been proposed that SHBG regulates the bioavailability of sex hormones by binding to circulating sex hormones (93). SHBG can also act as an active regulator of the steroid signaling system in target cells. Several epidemiological studies have consistently shown that high levels of SHBG in the blood are associated with a reduced risk of endometrial cancer in postmenopausal women (94–96).

As for estrogen, a MR study published in 2016 in women of European ancestry indicated and examined a positive association between estradiol and increased risk of EC, and identified *CYP19A1* as the main influencing gene (41). This study confirmed the association between EC and *CYP19A1* gene variants at the genome-wide level, and also provided evidence that the same group of variants was associated with higher concentrations of estradiol in postmenopausal women, supporting a causal role of estradiol in EC (41). Notably, the association was stronger in women with higher BMIs, suggesting that biologically, a gene-environment interaction seems plausible (41). Recently, the MR study of estradiol and cancer conducted by Larsson et al. also pointed out that a genetically predicted higher endogenous 17β estradiol concentration is associated with an increased risk of EC. Subtype analysis showed that endogenous 17β estradiol had the similar effect in endometrioid EC (56). In terms of male hormones, the results of a MR study published in 2020 on the effects of testosterone on disease in men and women (used only sex-specific genetic predictors as instrumental variable in EC analysis) showed that testosterone increased the risk of EC (mainly total EC and endometrioid EC) (48). In addition, the study notes that there was also evidence for a protective effect of SHBG on risk of EC in women (48). SHBG is negatively correlated with total testosterone and bioavailable testosterone in women, thus this study performed additional MR analysis using cluster-filtered testosterone variants to reduce confounding from SHBG. This analysis method can identify a subset of testosterone variants that do not depend on changes in SHBG. This effectively reduces the potential direct biological effects of SHBG and its confusion with obesity and insulin resistance, although cluster-filtered testosterone variants may still have secondary effects in SHBG levels (97). Similarly, an MR result performed by Mullee et al. showed that higher circulating total testosterone and free testosterone concentrations are associated with a higher risk of endometrial cancer, while SHBG is the opposite (55).

### Causality Between Age at Menarche or Menopause and EC Risk

For the endometrium, estrogen is the main stimulus for the proliferation of the endometrium, and the uncontrolled proliferation of the endometrium can lead to its malignant transformation (98–100). Therefore, estrogen is the cause and prerequisite for the development of at least some EC. There is evidence that long-term exposure to sex hormones such as estrogen may cause cancer of the reproductive organs (80, 101). Menarche is considered to be a sign of the beginning of ovulation, the beginning of changes in sex hormones in a woman’s body (102). Menopause is the end of female reproductive life. Women with early menarche time and later menopause time have higher levels of hormones and have a longer life-long exposure to estrogen (101, 103). Meanwhile, some research results support the hypothesis that the age at late menarche is negatively correlated with the risk of EC, and the age at menopause is positively associated with the risk of endometrial cancer (98, 102, 104).

For MR research, in 2017, Day et al. published a study on the timing of adolescence and cancer risk. This study identified hundreds of variants associated with age at menarche through genomic analysis, and concluded that increasing age at menarche adjusted for genetically predicted BMI was associated with lower risks for EC by using MR method (42). Furthermore, the MR study of O’Mara et al. in 2021 pointed out that the genetically predicted later menarche time is related to the lower risks of total EC and endometrioid EC (58). It is worth mentioning that the study did not find a statistically significant association between the genetically predicted later menopause time and the increased risk of EC (58).

### Causality Between Gynecological Diseases and EC Risk

Uterine fibroids, endometriosis and polycystic ovary syndrome are three common non-cancerous gynecological diseases affecting 5–69% (105), 10–15% (106) and 6–9% (107) of women of reproductive age, respectively. These non-cancerous gynecological diseases mainly affect premenopausal women, while endometrial cancer is mainly a postmenopausal malignant tumor. Even so, the two groups share some commonalities in risk factors (eg, inflammation, insulin resistance, chronic estrogen exposure, and obesity) (108–110). Many studies use observational data to assess the association between the three non-cancerous gynecological diseases and the risk of endometrial cancer. Unfortunately, the research results are heterogeneous (108–113). Because of this, Kho et al. conducted a study to explore the association of three gynecological diseases with EC at the genetic level. This study provides genetic evidence for a causal relationship between uterine fibroids and endometrial cancer. They also provided further evidence that the comorbidities of endometrial cancer, polycystic ovary syndrome, and uterine fibroids may be partly due to the genetic structure shared between these diseases (57).

### Causality Between Type 2 Diabetes and EC Risk

Type 2 diabetes is characterized by hyperglycemia, insulin resistance and inadequate secretion of insulin, each of which plays a role in the pathogenesis of EC (114–116). Studies have shown that IGF-1 also plays a role in diabetes. Elevated levels of IGF-1 are not a characteristic of type 2 diabetes, IGF-1 has been suggested to be protective against type 2 diabetes instead (117). Several epidemiological studies support a positive association of EC with hyperinsulinemia and type 2 diabetes (118–120). Estrogen-induced cyclic changes in IGF-1 expression and signaling modulate endometrial proliferation during the normal menstrual cycle. Increased expression of insulin and IGF-1 receptors are observed in endometrial hyperplasia, which heightens the responsiveness of these cells to insulin and IGF-1 and promotes hyperactivity of MAPK and PI3K/AKT/mTOR signaling frequently observed in EC. Proliferative signaling is further amplified by the loss of the PTEN tumor suppressor gene, which is an early event in the pathogenesis of EC. Finally, hyperglycemia due to insulin insensitivity helps to further promote the growth of metabolically active tissues, including endometrial hyperplasia and cancer (121, 122). The results of a meta-analysis also showed that there is an association between type 2 diabetes and endometrial cancer (123).

A 2015 MR study of type 2 diabetes, insulinemia, and EC, by Nead et al., showed that genetically predicted higher fasting insulin levels were associated with greater risk of EC (39). In addition, genetically predicted higher 30-minute post-challenge insulin levels were also associated with EC risk (39). However, no causal association was found between type 2 diabetes or fasting glucose and EC risk (39). After accounting for the potential confounding role of BMI, high insulin levels are still associated with an increased risk of EC (39). In 2020, Yuan et al. also conducted a MR study on Type 2 diabetes and cancer risk, which used 399 SNPs as instrumental variables for Type 2 diabetes to analyze data from UK Biobank, and obtained different results from Nead et al. Their results showed that genetically predicted type 2 diabetes was associated with an increased risk of EC (49).

### Causality Between Telomere Length and EC Risk

Telomeres are the protective structures at the ends of linear chromosomes and are regulated by many related proteins. The disruption of the regulatory network can disrupt the homeostasis of telomere length and lead to telomere dysfunction (that is, shorter or longer) and human diseases (124). When telomeres become dysfunctional, genomic instability ensues (125). The vast majority of cells undergo apoptosis, although a few cells may survive and be tumorigenic (125). Some studies found that compared with adjacent normal endometrial tissue, the telomere length of endometrial tumor tissue was shortened (126–128), whereas another did not find significant differences in length between adjacent normal, endometrial hyperplasia and EC (129). However, a recent MR study published in 2017 using 12 SNPs as instrumental variables on subjects of European descent showed a significant causal relationship of longer telomere length with increased risk of EC (43).

### Causality Between Selenium and EC Risk

Selenium is an important trace element in the human body that individuals are exposed to mainly through food consumption, although exposure can also occur through air, drinking water, and dietary supplements (130). Selenium is an important component of selenoproteins and plays a key role in anti-oxidative stress (131). From the late 1960s, a few observational studies reported that people with high levels of selenium in their diet or in their body tissues had lower risk of cancer (132). Moreover, some laboratory studies showed that selenium could inhibit the growth of cancer cells (132). Although, RCTs have shown that selenium supplementation has no benefit in reducing the risk of cancer (133), a recent meta-analysis of the association between selenium intake (diet and supplementation) and overall cancer risk showed that people with higher selenium intake have a lower incidence of cancer (134). In general, the answer to the question of whether selenium has anti-cancer effects is always controversial. For EC, there are no RCT/limited observational studies investigating the impact of selenium on EC risk (135). Thus, it is still unclear about the effect of selenium on EC. Similar to some observational studies, the results of an MR study published in 2020 pointed out that there is no causal association between selenium and EC risk (44).

### Causality Between Cortisol and EC Risk

Cortisol is a glucocorticoid that plays a vital role in the body’s physiological response to endogenous and exogenous stress. However, some evidence suggests that cortisol may be related to the development of cancer. The immunosuppressive effect of cortisol may lead to a decrease in the immune surveillance of early cancer, promote its immune escape and acquire further cancer-causing mutations (136, 137). In addition, cortisol has the effect of causing obesity and hyperglycemia. Weight gain and insulin resistance are all related to the increased risk of a series of malignant tumors (63). However, there are still relatively few epidemiological data related to cortisol itself and cancer risk. In order to understand whether cortisol increases the risk of cancer, Larsson et al. conducted an MR randomization study on cortisol and cancer. The results indicate that elevated plasma cortisol levels may increase the risk of endometrial cancer but not other cancers (54).

### Causality Between Coffee Consumption and EC Risk

Coffee is one of the most widely consumed beverages in the world, so any benefits of coffee to human health may have a significant impact on public health. In animal experiments, these active compounds derived from coffee (such as caffeine, flavonoids, lignans and other polyphenols) have been shown to increase energy expenditure, regulate DNA repair-related genes, and inhibit chronic inflammation (138, 139). Antioxidant compounds in coffee beans, such as chlorogenic acid, kahweol, and cafestol are considered to have anticarcinogenic properties (138, 140). At the same time, a number of epidemiological studies on coffee and cancer have also pointed out the protective effect of coffee on EC (141–143). Unfortunately, the MR study on coffee and cancer by Ong et al. did not find a similar association between the two (45).

## Discussion

Traditional risk factors have often been linked to endometrial cancer through observational studies, and some have been further assessed through interventional studies. Observational studies, also called epidemiologic study, are mostly retrospective and assess the underlying causality of exposure-outcome relationships that influence prevention approaches; interventional studies are usually prospective and designed specifically to assess the direct effect of treatment or prevention on disease (144). Both include three elements: 1) definition and measure of exposure in two or more groups, 2) measure of health outcome(s) in these same groups, and 3) statistical comparison made between groups to assess potential relationships between the exposure and outcome (145). Each study design has specific outcome measures that rely on the type and quality of data utilized. In addition, different research methods also have their own limitations, and it is necessary to expand research methods to improve them. MR analysis is gradually becoming an effective tool for epidemiological research. MR analysis is suitable for studying the following associations between the following exposure and risks of EC. First, physical characteristics (height, weight, etc.), these characteristics are often not easy to intervene; Second, long-term exposures (coffee, tea, etc.), this type of exposure often lasts for a long time, and the cost and time spent on RCT are too high; Third, harmful exposures (cigarettes, drugs, etc.), past studies have shown that such exposures may cause adverse effects, and RCT on them is against ethics and morality.MR analysis uses SNP as an instrumental variable to explore the relationship between these exposures and results, which is very suitable for studying this type of exposure.

Another advantage of MR research is that in the face of certain closely related exposures, it can adjust instrumental variables from a genetic perspective, making the results more reliable. As a common risk factor for EC, BMI is a basic indicator of the human body. When we use other methods to understand the relationship between exposure and outcome, it is difficult to rule out the influence of BMI on the results. In MR research, we can isolate certain exposures by adjusting instrumental variables, thereby obtaining more targeted results. This advantage is also reflected in the study of sex hormones and EC risks. For example, SHBG plays an important role in the physiological process of testosterone. It is not easy to distinguish between the two in other research methods, which may affect the results. MR research can analyze the two separately to make the results more reliable. For other experiments (observational research, cell/animal experiments), it is a very instructive supplement and explanation. However, because of the differences in the selection of SNPs as instrumental variables, theresults of MR analysis may be different when exploring the same exposure.

According to the classification of risk factors, we have sorted out the MR researches and their results related to EC from 2015 to the present in detail. Readers can directly and comprehensively understand the application of MR research in the field of EC by reading the present review. In addition, when comparing the results of MR analysis and other research methods, we have the following findings. In terms of obesity and obesity-related factors, MR analysis and other studies on the effects of adiponectin, leptin and TNF on EC risk have obtained different results. The results of MR analysis showed that adiponectin and leptin had no effect on EC risk, while higher TNF concentration was related to lower EC risk. There are many studies on the association between EC risk and obesity or obesity-related factors. The results obtained by various research methods are not the same. Based on the strong correlation between these exposures, we can consider using multivariable MR method to conduct more in-depth research on some of the key exposures (146).

Similarly, the results of MR analysis that differ from other designed studies are also reflected in height, menopausal time, and coffee consumption. MR analysis did not find the association between aforementioned exposures and EC risk changes. It is worth mentioning that although the results of MR analysis are different from other research methods, we failed to observed the opposite findings among these included studies. In addition to the weak correlation between exposure and outcomes at the genetic level, the reason for this phenomenon may also be due to the insufficient selection of instrumental variables and different research objects. Two included studies on T2D reported different results. The MR study published by Nead et al. in 2005 on the British and Australian populations did not find an association between T2D and EC. However, the study by Yuan et al. (data source: UK Biobank) in 2020 confirmed that T2D is associated with a higher risk of EC. In addition to the different data sources, the different selection of instrumental variables might be the major reason (Nead et al. used 49 SNPs, Yuan et al. used 399 SNPs). This also shows that when conducting MR research on EC, it is not only necessary to repeatedly improve the screening process of instrumental variables, but also to consider the research object. It is also necessary to conduct MR studies in different populations (Europeans, Asians, Africans, etc.). In addition, MR analysis can also be performed on some risk factors that are less reported by EC (such as cortisone). This approach not only saves costs, but also guides follow-up research. It can even predict the results of RCT of drugs and increase the success rate of drug development (147).

Meanwhile, the power considerations in MR research are also worthy of attention. For example, Kho et al. selenium MR study did not support a causal relationship between selenium level and endometrial cancer because instrumental variables only capture a small proportion of trait variance. Limited power to detect associations due to small proportion of trait variance captured by instrumental variables is often the explanation of null findings in MR studies. Therefore, proportion of trait variance captured by instrumental variable should be considered when MR studies fails to support a causal relationship. GWAS is constantly evolving, and more specific and accurate exposure-related SNPs can be successfully identified. Using these SNPs as instrumental variables, with the enrichment of statistical methods and the deepening of observational research, the results of MR analysis will be more accurate and reliable. Therefore, even for MR studies with the same exposure and outcome, it is also necessary to conduct again after adjusting the IVs.

In addition, MR research is ultimately to explore the potential cause and effect of exposure and outcome at the genetic level, which have not considered the clinical importance of magnitude of the potential causal effects. Although currently available MR studies have prioritized several risk factors of EC, it is unclear if the potential causal effects size derived from MR studies indicate a clinically important difference in the outcome (148). Thus, findings from MR studies still require more work (e.g., RCT if it is feasible to conduct) to test if the intervention has a clinically important effect on the outcome. From another perspective, the results of the MR study do not only reveal potential causal associations. It can be used as a new perspective to verify the results of past experiments, and it can also serve as a guide and reminder for subsequent scientific research. Combining research methods (such as RCT, observational research, cell/animal experiments, etc.) with MR research may complement and promote each other, thus making the research road wider and wider.

Currently, there have been some constructive findings on EC risk factors, especially obesity and related biomarkers (149). A variety of rational interventions to prevent EC are also being investigated and applied, including potential lifestyle interventions and surgical procedures that decrease visceral adiposity, as well as medications that aim to interrupt or reverse the hormonal and metabolic derangements associated with obesity and insulin resistance (11, 150, 151). For EC, we should make more connections between observational and interventional studies. The two can be used as a reference for each other to interpret and guide the research, allowing us to have a more comprehensive and in-depth understanding of EC. This is conducive to the formation of a more complete prevention and treatment strategy for EC.

## Conclusion

In conclusion, MR analysis plays an important role in etiological research on EC. Overall, type 2 diabetes, uterine fibroids, higher BMI, higher PAI-1, higher fasting insulin, early insulin secretion, longer telomere length, higher testosterone and higher plasma cortisol levels are associated with increased risk of EC. Conversely, later age of menarche, higher circulatory TNF, LDL cholesterol, and SHBG levels are associated with reduced risk of EC. Although there are some limitations, MR analysis can still provide constructive insights in drug development and disease prevention, and provide effective guidance for observational research and RCT.

## Author Contributions

J-ZG, Q-JW, T-TG, and GL contributed to the study design. J-ZG collection and analysis of data. J-ZG, Q-JW, F-HL, CG, T-TG, and GL wrote the first draft of the manuscript and edited the manuscript. All authors read and approved the final manuscript.

## Funding

This work was supported by the Natural Science Foundation of China (No. 82073647 to Q-JW, No. 81602918 to Q-JW, and No.82103914 to T-TG), LiaoNing Revitalization Talents Program (No. XLYC1907102 to Q-JW), Shenyang high level innovative talents support program (No. RC190484 to Q-JW), and 345 Talent Project of Shengjing Hospital of China Medical University (Q-JW and T-TG).

## Conflict of Interest

The authors declare that the research was conducted in the absence of any commercial or financial relationships that could be construed as a potential conflict of interest.

## Publisher’s Note

All claims expressed in this article are solely those of the authors and do not necessarily represent those of their affiliated organizations, or those of the publisher, the editors and the reviewers. Any product that may be evaluated in this article, or claim that may be made by its manufacturer, is not guaranteed or endorsed by the publisher.
